# Enaminones as Building Blocks for the Synthesis of Substituted Pyrazoles with Antitumor and Antimicrobial Activities

**DOI:** 10.3390/molecules16021834

**Published:** 2011-02-22

**Authors:** Sayed M. Riyadh

**Affiliations:** Department of Chemistry, Faculty of Science, University of Cairo, Giza, 12613, Egypt; E-Mail: riyadh1993@hotmail.com

**Keywords:** (dimethylamino)acryloylpyrazole, hydrazonoyl chlorides, antitumor activity, antimicrobial activity

## Abstract

Novel *N*-arylpyrazole-containing enaminones **2a,b** were synthesized as key intermediates. Reactions of **2a,b** with active methylene compounds in acetic acid in the presence of ammonium acetate afforded substituted pyridine derivatives **5a-d**. Enaminones **2a,b** also reacted with aliphatic amines such as hydrazine hydrate and hydroxylamine hydrochloride to give bipyrazoles **8a,b** and pyrazolylisoxazoles **9a,b**, respectively. On the other hand, treatment of **2a,b** with a heterocyclic amine and its diazonium salt yielded the respective [1,2,4]triazolo[4,3-*a*]pyrimidines **12a,b** and pyrazolylcarbonyl[1,2,4]triazolo-[3,4-*c*][1,2,4]triazines **14a,b**. Moreover, 2-thioxo-2,3-dihydro-1*H*-pyrido[2,3-*d*]pyrimidin-4-one (**17**) was prepared *via* reaction of enaminone **2a** with aminothiouracil (**15**). Cyclocondensation of **17** with the appropriate hydrazonoyl chlorides **18a-c** gave the corresponding pyrido[2,3-*d*][1,2,4]triazolo[4,3-*a*]pyrimidin-5-ones **21a-c**. The cytotoxic effects of compounds **2b**, **14a **and **17** against human breast cell line (MCF-7) and liver carcinoma cell line (HEPG2) were screened and in both lines they showed inhibition effects comparable to those of 5-fluorouracil, used as a standard. The antimicrobial activity of some products chosen as representative examples was also evaluated.

## 1. Introduction

**Figure 1:**
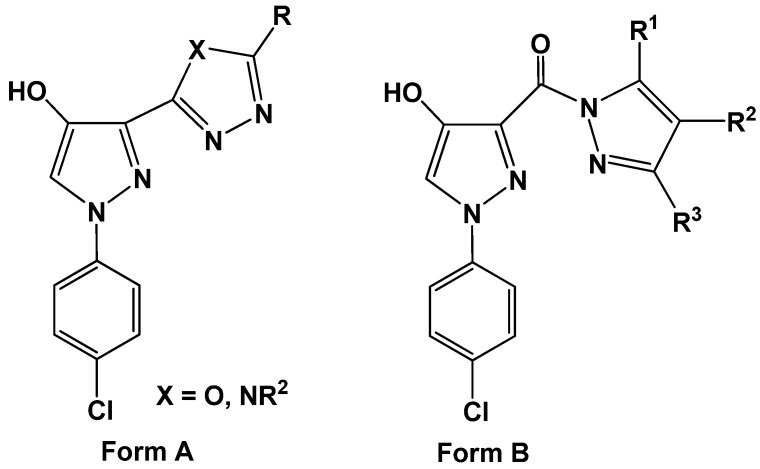
substituted pyrazoles with potential antitumor activity.

The growing interest in bioactive *N*-arylpyrazoles has led to an increasing demand for efficient syntheses of this class of heterocyclic compounds. Several reports have found diverse applications for *N*-arylpyrazoles in medicine such as antitumor [1,2,3,4,5,6,7,8,9,10,11], antiviral [12], anti-inflammatory [13] agents, or kinase inhibitors for the treatment of type 2 diabetes, hyperlipidemia, and obesity [14]. Moreover, these compounds have remarkable potential in nanomedicine applications against malignant gliomas [15]. 1-(4-Chlorophenyl)-4-hydroxy-3-substituted-1*H*-pyrazoles (Figure 1) were reported by the U.S. National Cancer Institute (NCI) to have pronounced anticancer activity [16,17]. 

Structure modifications suggested in this work focused mainly on synthesis of polysubstituted pyrazole analogues to form **A** and **B**, having a variety of azoles and fused azoles at position 3. These substituents at position 3 are linked directly to the pyrazole ring or through the carbonyl group in order to improve the antitumor and antimicrobial activities of such compounds. This work is an extension of an ongoing research program devoted to the synthesis and characterization of different heterocyclic ring systems endowed with potential biological activities [18,19,20,21,22,23,24,25].

## 2. Results and Discussion

**Scheme 1:**
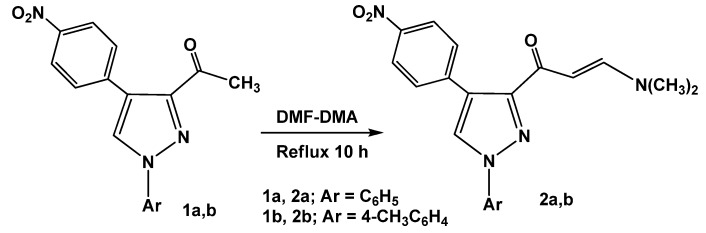
Synthesis of 3-[*E*-3-(*N*,*N*-dimethylamino)acryloyl]-4-(4-nitrophenyl)-1-aryl-1*H*-pyrazoles (**2a,b**).

The synthetic route for preparation of the previously unreported 3-[*E*-3-(*N*,*N*-dimethyl-amino)acryloyl]-4-(4-nitrophenyl)-1-aryl-1*H*-pyrazoles (**2a,b**), involving condensation of 3-acetyl-4-(4-nitrophenyl)-1-aryl-1*H*-pyrazoles (**1a,b**) [26] with dimethylformamide dimethylacetal (DMF-DMA) under reflux for 10 hours in the absence of solvent, is depicted in Scheme 1. 

The structures of **2a,b** were confirmed by their spectral data (IR, MS and ^1^H-NMR) and elemental analyses. For example, the ^1^H-NMR spectrum revealed two doublet signals at *δ* 5.88, 7.67 ppm with coupling constant *J* = 13 Hz assignable to olefinic protons (CH=CH) in a *trans* configuration [26,27] besides two singlet signals of the dimethylamino group at *δ* 2.8, 3.1 ppm.

**Scheme 2:**
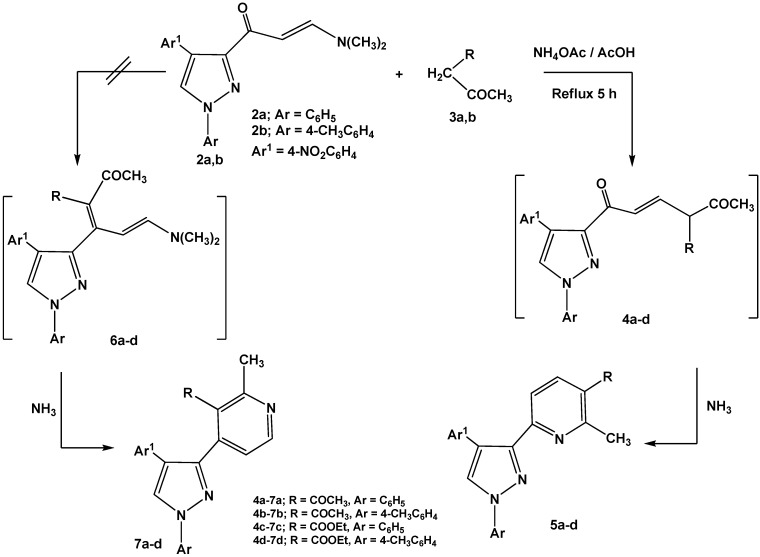
Reactions of enaminones **2a,b** with active methylene compounds.

Reactions of enaminones **2a,b** with *C*-nucleophiles such as 2,4-pentanedione and ethyl 3-oxo-butanoate were carried out in glacial acetic acid in the presence of ammonium acetate and led to formation of 6-(pyrazol-3-yl)-pyridine derivatives **5a-d** *via* nucleophilic displacement of active methylene to the dimethylamino group followed by concurrent elimination of water molecule from non-isolable intermediates **4a-d** (Scheme 2). The other possible isomeric structures 4-(pyrazol-3-yl)-pyridines **7a-d** were discarded based on ^1^H-NMR data that revealed pyridyl hydrogens at C-4, C-5 as a pair of doublets at *δ* 7.5, 7.7 ppm, respectively, with *J* = 8 Hz assignable to 6-substituted-pyridines **5a-d**. The isomeric structures **7a-d** should display pair of doublets corresponding to C-5, C-6 with a lower coupling constant (*J* = 2–3 Hz) [28].

**Scheme 3:**
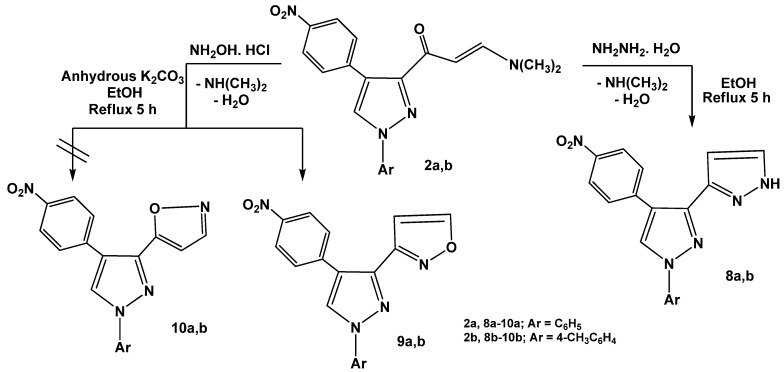
Reactions of enaminones **2a,b** with hydrazine hydrate and hydroxylamine.

Treatment of enaminones **2a,b** with a *N*-nucleophile such as hydrazine hydrate in absolute ethanol under reflux afforded 1*H*,1'*H*-3,3'-bipyrazoles **8a,b**. The structures of the products were substantiated by the ^1^H-NMR spectra which displayed new pair of doublets at *δ* 7.53 and 7.58 ppm with (*J* = 7.5 Hz) corresponding to pyrazole protons at positions 4 and 5, respectively and another D_2_O exchangeable proton at *δ* 13 ppm assignable to the NH group. The products were formed *via* initial addition of the amino group in hydrazine to the enaminone double bond, followed by elimination of dimethylamine and water molecules to give the final isolable products **8a,b **as previously mentioned [29] (Scheme 3). Similarly, enaminones **2a,b** reacted with hydroxylamine hydrochloride in refluxing absolute ethanol in the presence of anhydrous potassium carbonate to yield products that may be formulated as pyrazolylisoxazoles **9a,b** or its isomeric forms **10a,b**. Structure **9 **was assigned for the reaction products on the basis of the ^1^H-NMR spectral data in which a resonance for H-4 and H-5 of isoxazole appeared typically at *δ* 6.78 and 8.50 ppm, respectively (see Experimental). The other isomeric structures **10a,b** were ruled out as the isoxazole H-3 would be expected to resonate at a higher field of *δ* 8.0 ppm [30]. It is thus assumed that, the products **9a,b** were formed *via* initial condensation of amino group of hydroxylamine with carbonyl group of enaminones **2a,b** followed by elimination of dimethylamine (cf. Scheme 3). 

**Scheme 4:**
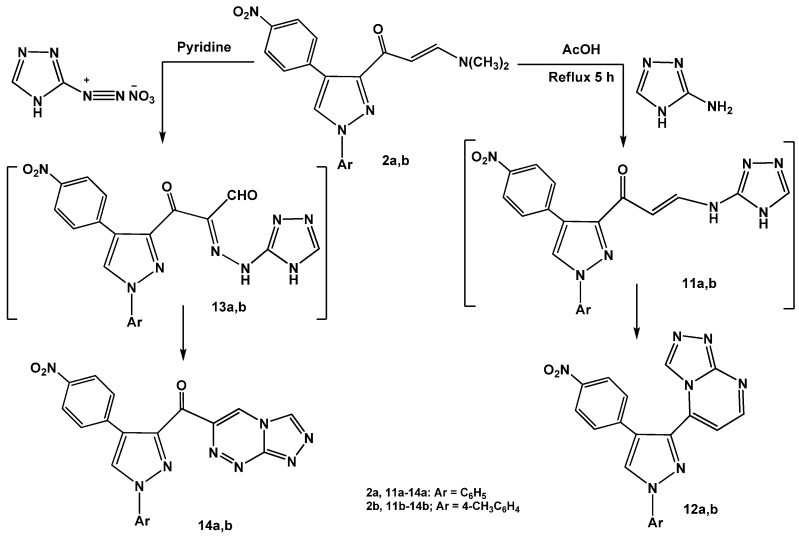
Reactions of enaminones **2a,b** with heterocyclic amines.

Next, the reactions of enaminones **2a,b** with heterocyclic amines were investigated. Refluxing of enaminones **2a,b** with 3-amino-1*H*-[1,2,4]triazole in glacial acetic acid gave the corresponding [1,2,4]triazolo[4,3-*a*]pyrimidines **12a,b** *via* non-isolable intermediates **11a,b** (Scheme 4). 

The structures of the products were confirmed by spectral (IR, MS and ^1^H-NMR) and elemental analyses (see Experimental). On the other hand, coupling of enaminones **2a,b** with diazotized 3-amino-1*H*-[1,2,4]triazole in pyridine at low temperature afforded the respective pyrazolylcarbonyl- [1,2,4]triazolo[3,4-*c*][1,2,4]triazines **14a,b**. The reactions proceeded by initial formation of non-isolable hydrazonals [31,32,33] **13a,b** followed by elimination of water molecules to give the desired products **14a,b**. 

The utility of enaminone **2a** in the synthesis of annelated heterocycles was further explored *via* its reaction with 6-amino-2-thioxo-2,3-dihydropyrimidin-4(1*H*)-one (**15**) in glacial acetic acid under reflux for 6 hours. This reaction afforded the 2-thioxo-2,3-dihydro-1*H*-pyrido[2,3-*d*]pyrimidin-4-one** 17** *via* intermediate **16**. Spectral (IR, MS, ^1^H-NMR) data and elemental analysis were in consistent with the isolated product **17**.

**Scheme 5:**
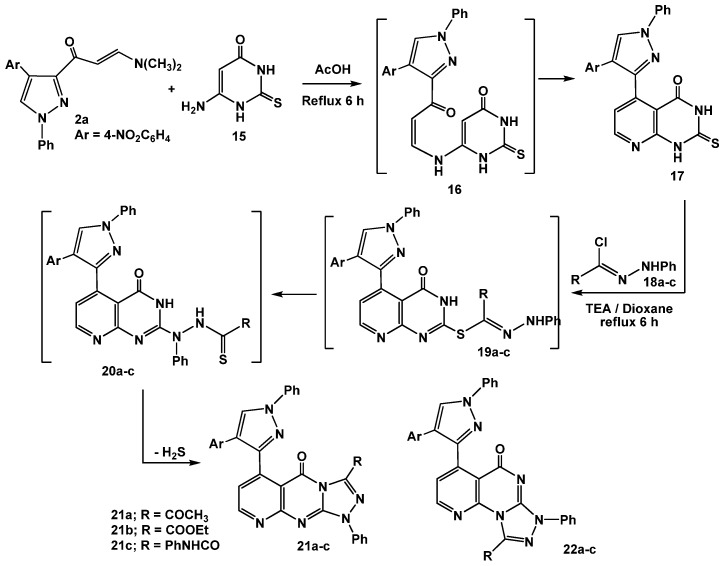
Reactions of enaminone **2a** with pyrimidinethione.

For example, IR revealed three absorption bands at 3261, 3245, 1677 cm^−1^ assignable for 2 NH, and a C=O, respectively. The ^1^H-NMR spectrum also displayed a characteristic pair of doublet signals at *δ* 8.29, 8.48 ppm assigned to the pyridine H-2, H-3 protons, respectively [34]. Treatment of 2-thioxo-2,3-dihydro-1*H*-pyrido[2,3-*d*]pyrimidin-4-one (**17**) with the appropriate hydrazonoyl chlorides **18a-c** in dioxane in the presence of triethylamine under reflux conditions furnished the corresponding pyrido[2,3-*d*][1,2,4]triazolo[4,3-*a*]pyrimidinones** 21a-c **as the end products (Scheme 5). 

**Figure 2:**
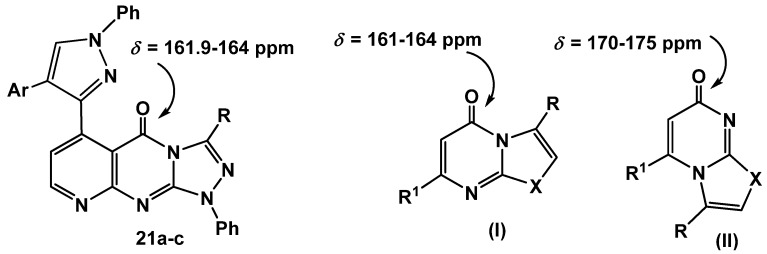
^13^C NMR for azolopyrimidinones.

The reactions proceeded through *S*-alkylation [35] to give *S*-alkylated products **19a-c** followed by Smiles rearrangement [36], affording intermediates **20a-c** which cyclized *in situ* under the employed reaction conditions *via* elimination of hydrogen sulfide gas to give the desired products **21a-c **(cf. Scheme 5). The other isomeric structures, pyrido[2,3-*d*][1,2,4]triazolo[3,4-*a*]pyrimidinones** 22a-c**, were ruled out based on the ^13^C-NMR which revealed a signal for a carbonyl group at *δ* = 161.9–164 ppm which is similar to that of **I **(*δ* = 161–164 ppm) and different from its isomeric structure **II **(*δ* = 170–175) [37] (Figure 2).

### 2.1. Antitumor Screening Test

**Figure 3:**
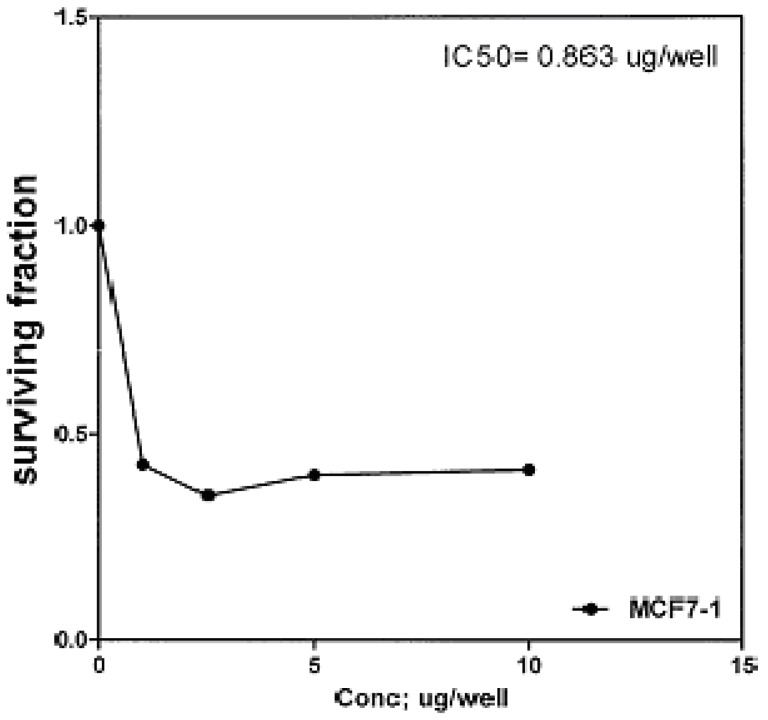
Effect of conc. of **2b** on MCF-7 line.

**Figure 4:**
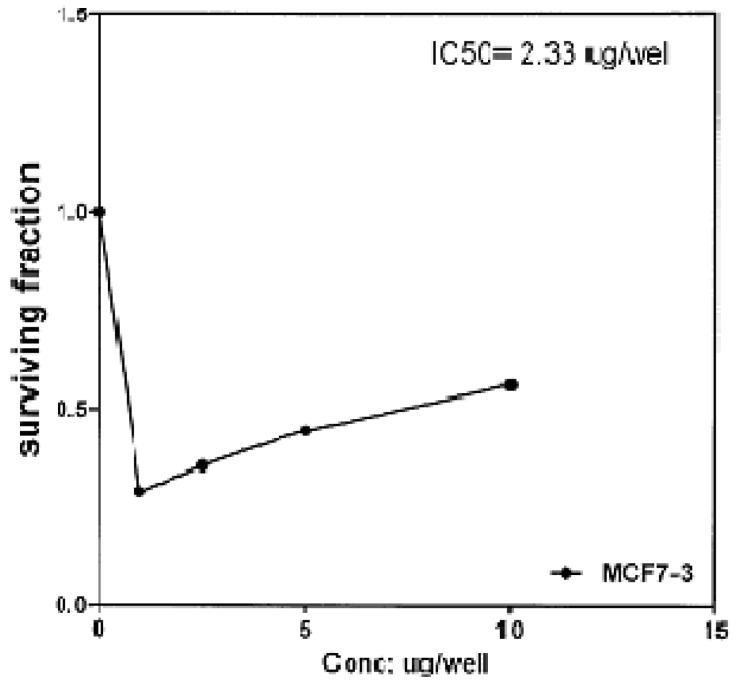
Effect of conc. of **14a** on MCF-7 line.

**Figure 5:**
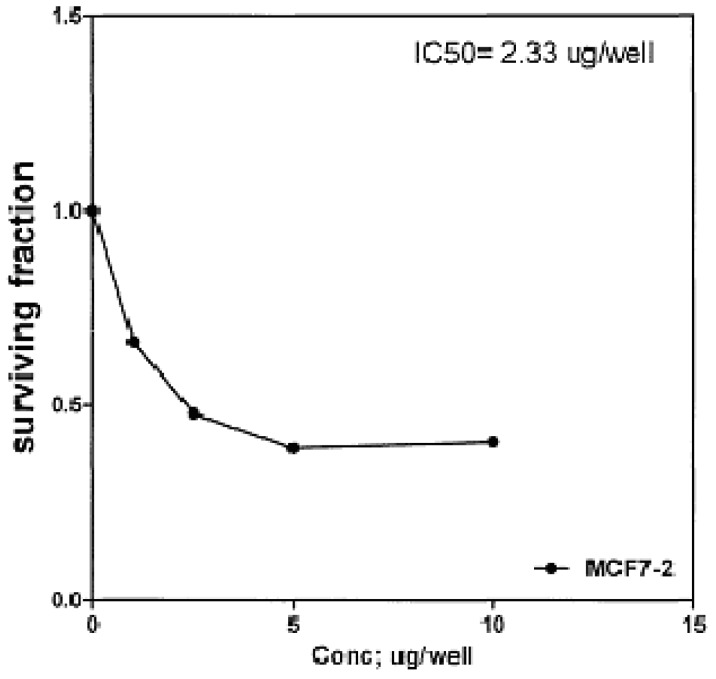
Effect of conc. of **17** on MCF-7 line.

**Figure 6:**
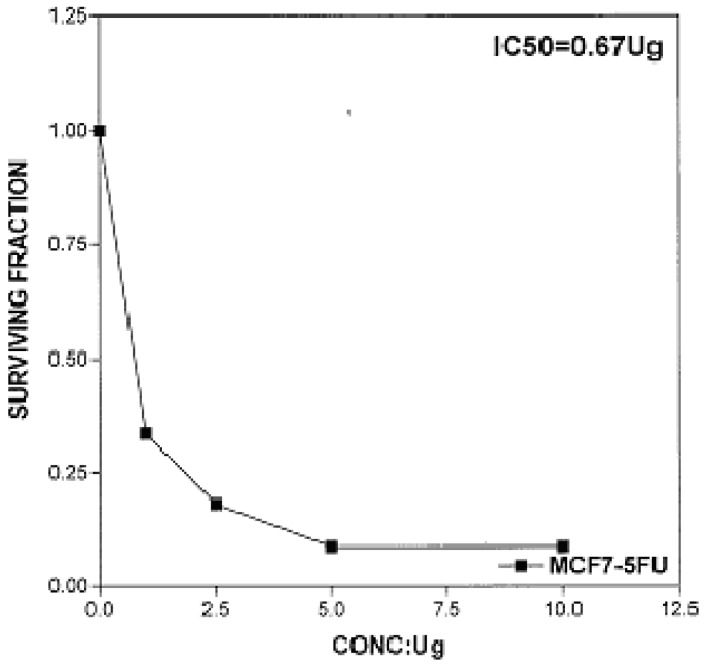
Effect of conc. of 5-fluorouracil (standard) on MCF-7 line.

The cytotoxic effects of compounds **2b**, **14a** and **17** against human breast cell line (MCF-7) and liver carcinoma cell line (HEPG2) were evaluated using 5-fluorouracil as a standard sample in both lines. These compounds were selected by the National Cancer Institute (NCI), Cairo, Egypt. The analysis of the data obtained indicated that the values of IC_50_ for such compounds against human breast cell MCF-7 line are 0.863 μg/well (Figure 3), 2.33 μg/well (Figure 4), and 2.33 μg/well (Figure 5), respectively [IC_50_ of 5-fluorouracil as a standard sample = 0.67 μg] (Figure 6). The results indicated that biologically active compound **2b** has almost the same activity as the reference drug (5-fluorouracil).

**Figure 7:**
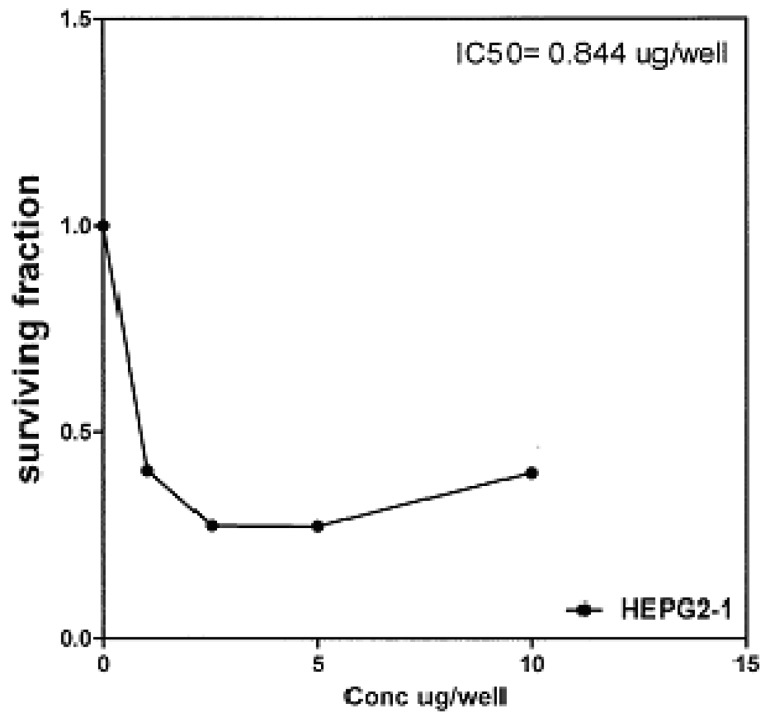
Effect of conc. of **2b** on HEPG2 line.

**Figure 8:**
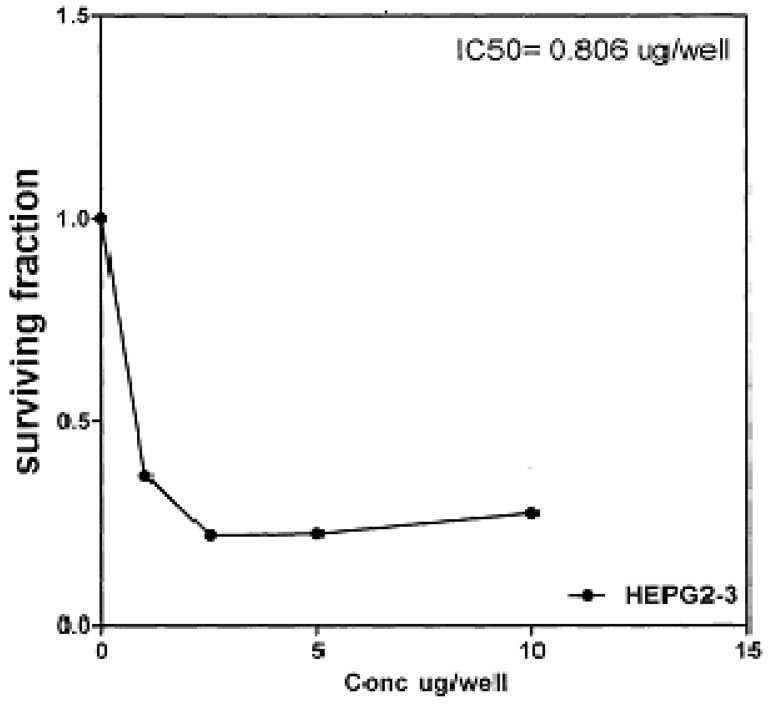
Effect of conc. of **14a** on HEPG2 line.

**Figure 9:**
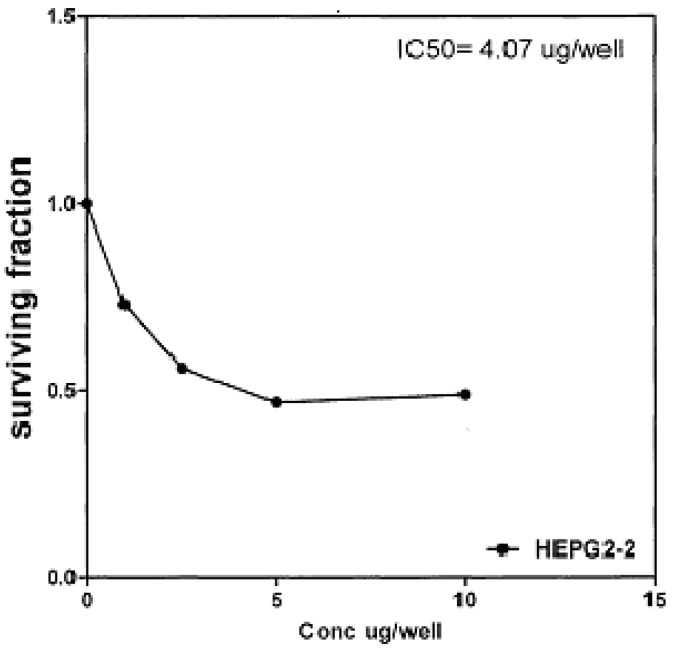
Effect of conc. of **17** on HEPG2 line.

**Figure 10:**
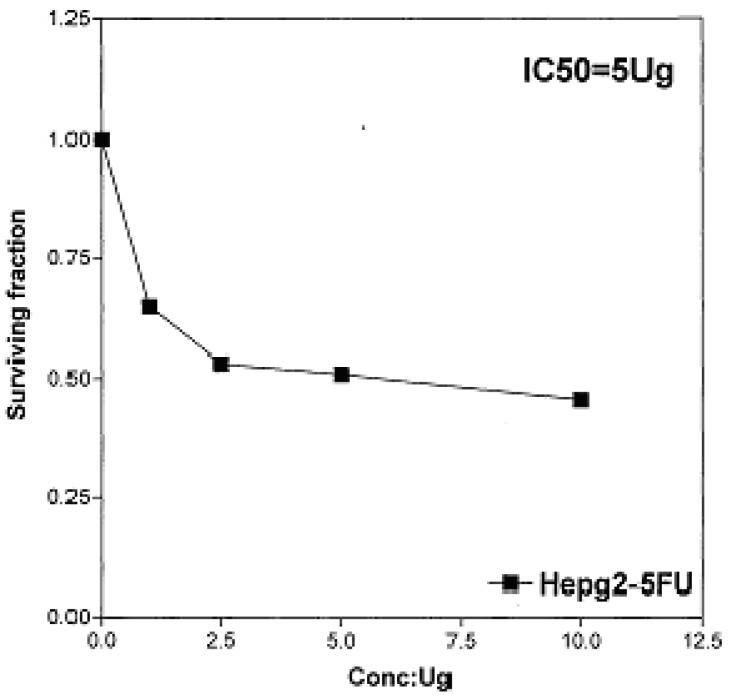
Effect of conc. of 5-fluorouracil (standard) on HEPG2 line.

On the other hand, IC_50_ of compounds **2b, 14a **and **17** against liver carcinoma cell line (HEPG2) are 0.884 μg/well (Figure 7), 0.806 μg/well (Figure 8), and 4.07 μg/well (Figure 9), respectively. [IC_50_ of 5-fluorouracil as standard sample = 5 μg] (Figure 10). The values of IC_50_ indicated that the tested compounds **2b, 14a **and **17** have higher cytotoxic activities against liver carcinoma cell line (HEPG2) than standard drug (5-fluorouracil). The cytotoxic activity was measured by the Skehan *et al.* method (see Experimental). 

The results of biological screening allow the following assumptions about the structure activity relationships (SAR) of these compounds:

- The presence of nitrogenous fused heterocycles at position 3 of the main pyrazole moiety, linked directly or through carbonyl group, with multicenters for hydrogen accepting properties are essential for activity where it can intercalate within the DNA strands.
- The 3-[*E*-3-(*N*,*N*-dimethylamino)acryloyl]-4-(4-nitrophenyl)-1-aryl-1*H*-pyrazoles **2a,b** are essential for antitumor activity.

### 2.2. Antimicrobial Activity

The newly synthesized products **2a**, **2b**, **5b**, **5c**, **8b**, **9b**, **12b**, **14a**, **21a** and **21b** were tested for their antimicrobial activities using four species of fungi, namely *Aspergillus fumigatus* **AF**, *Penicillium italicum* **PI**, *Syncephalastrum racemosum* **SR** and *Candida albicans* **CA**, in addition to four bacterial species, namely *Staphylococcus aureus* **SA**, *Pesudomonas aeruginosa* **PA**, *Bacillus subtilis* **BS** and *Escherichia coli* **EC**. The organisms were tested against the activity of solutions of three different concentrations [5 mg/mL, 2.5 mg/mL, 1.25 mg/mL] of each compound and using inhibition zone diameter (IZD) in mm as criterion for the antimicrobial activity. The fungicide *terbinafine* and the bactericide *chloramphenicol* were used as references to evaluate the potency of the tested compounds under the same conditions. The results, depicted in Table 1, Table 2, Table 3, revealed that compounds **2a **and **5c **exhibited high degree of inhibition against **SA**, and **BS**. Compounds **9b**, **12b**, **14a**, **21a **and **21b** have high inhibition effects against **AF**, **PI**, **SR **and **SA**. These compounds also exhibited moderate inhibition effect against **CA** and **BS**. All the tested compounds were reflecting no inhibition of growth against **PA** and **EC**.

**Table 1:** Antimicrobial activity of products **2a**, **2b**, **5b**, and **5c**.

(Sample)	2a (mg/mL)			2b (mg/mL)			5b (mg/mL)			5c (mg/mL)			Standard^*^		
**Tested Microorganism**	**5**	**2.5**	**1.25**	**5**	**2.5**	**1.25**	**5**	**2.5**	**1.25**	**5**	**2.5**	**1.25**	**5**	**2.5**	**1.25**
***Aspergillus fumigatus* (AF)**	**9**	**5**	**0**	**8**	**0**	**0**	**7**	**0**	**0**	**6**	**4**	**0**	**24**	**18**	**11**
***Penicillium italicum* (PI)**	**7**	**3**	**0**	**6**	**0**	**0**	**5**	**0**	**0**	**5**	**3**	**0**	**19**	**9**	**4**
***Syncephalastrum racemosum* (SR)**	**12**	**7**	**3**	**12**	**9**	**5**	**14**	**9**	**0**	**11**	**9**	**0**	**21**	**13**	**9**
***Candida albicans* (CA)**	**9**	**6**	**3**	**7**	**4**	**0**	**9**	**4**	**0**	**12**	**9**	**5**	**19**	**10**	**6**
***Staphylococcus aureus* (SA)**	**11**	**7**	**4**	**11**	**8**	**5**	**14**	**8**	**5**	**11**	**8**	**5**	**15**	**6**	**4**
***Pesudomonas aeruginosa* (PA)**	**0**	**0**	**0**	**0**	**0**	**0**	**0**	**0**	**0**	**0**	**0**	**0**	**11**	**5**	**0**
***Bacillus subtilis* (BS)**	**15**	**8**	**6**	**12**	**7**	**4**	**14**	**9**	**4**	**18**	**13**	**9**	**22**	**18**	**11**
***Escherichia coli* (EC)**	**0**	**0**	**0**	**0**	**0**	**0**	**0**	**0**	**0**	**0**	**0**	**0**	**27**	**20**	**13**

^*^Chloramphenicol was used as a standard antibacterial agent while, Terbinafine was used as a standard antifungal agent.

**Table 2:** Antimicrobial activity of products **8b**, **9b**, **12b**, and **14a**.

(Sample)	8b (mg/mL)			9b (mg/mL)			12b (mg/mL)			14a (mg/mL)			Standard^*^		
**Tested Microorganism**	**5**	**2.5**	**1.25**	**5**	**2.5**	**1.25**	**5**	**2.5**	**1.25**	**5**	**2.5**	**1.25**	**5**	**2.5**	**1.25**
***Aspergillus fumigatus*** ** (AF)**	**9**	**7**	**3**	**22**	**14**	**9**	**18**	**11**	**6**	**16**	**7**	**3**	**24**	**18**	**11**
***Penicillium italicum*** ** (PI)**	**10**	**6**	**3**	**14**	**6**	**3**	**13**	**6**	**4**	**0**	**0**	**0**	**19**	**9**	**4**
***Syncephalastrum racemosum*** ** (SR)**	**9**	**7**	**4**	**19**	**12**	**8**	**16**	**9**	**6**	**18**	**12**	**7**	**21**	**13**	**9**
***Candida albicans*** ** (CA)**	**10**	**8**	**4**	**9**	**6**	**2**	**10**	**7**	**3**	**9**	**6**	**2**	**19**	**10**	**6**
***Staphylococcus aureus*** ** (SA)**	**12**	**7**	**4**	**12**	**8**	**5**	**13**	**8**	**5**	**11**	**8**	**5**	**15**	**6**	**4**
***Pesudomonas aeruginosa*** ** (PA)**	**0**	**0**	**0**	**0**	**0**	**0**	**0**	**0**	**0**	**0**	**0**	**0**	**11**	**5**	**0**
***Bacillus subtilis*** ** (BS)**	**16**	**9**	**5**	**15**	**8**	**5**	**14**	**10**	**7**	**10**	**8**	**4**	**22**	**18**	**11**
***Escherichia coli*** ** (EC)**	**0**	**0**	**0**	**0**	**0**	**0**	**0**	**0**	**0**	**0**	**0**	**0**	**27**	**20**	**13**

^*^Chloramphenicol was used as a standard antibacterial agent while, Terbinafine was used as a standard antifungal agent.

**Table 3:** Antimicrobial activity of products **21a** and **21b.**

(Sample)	21a (mg/mL)			21b (mg/mL)			Standard^*^		
**Tested Microorganism**	**5**	**2.5**	**1.25**	**5**	**2.5**	**1.25**	**5**	**2.5**	**1.25**
***Aspergillus fumigatus* (AF)**	**20**	**11**	**6**	**19**	**14**	**9**	**24**	**18**	**11**
***Penicillium italicum* (PI)**	**17**	**6**	**4**	**13**	**6**	**3**	**19**	**9**	**4**
***Syncephalastrum racemosum* (SR)**	**15**	**8**	**6**	**17**	**12**	**8**	**21**	**13**	**9**
***Candida albicans* (CA)**	**10**	**7**	**3**	**9**	**6**	**2**	**19**	**10**	**6**
***Staphylococcus aureus* (SA)**	**13**	**7**	**5**	**10**	**8**	**5**	**15**	**6**	**4**
***Pesudomonas aeruginosa* (PA)**	**0**	**0**	**0**	**0**	**0**	**0**	**11**	**5**	**0**
***Bacillus subtilis* (BS)**	**12**	**9**	**7**	**13**	**10**	**8**	**22**	**18**	**11**
***Escherichia coli* (EC)**	**0**	**0**	**0**	**0**	**0**	**0**	**27**	**20**	**13**

^*^Chloramphenicol was used as a standard antibacterial agent while, Terbinafine was used as a standard antifungal agent.

## 3. Experimental

### 3.1. General

All melting points were determined on an electrothermal Gallenkamp apparatus and are uncorrected. Solvents were generally distilled and dried by standard literature procedures prior to use. The IR spectra were measured on a Pye-Unicam SP300 instrument in potassium bromide discs. The ^1^H NMR spectra were recorded on a Varian Mercury VXR-300 spectrometer (300 MHz) and the chemical shifts were related to that of the solvent DMSO-d_6_. The mass spectra were recorded on a GCMS-Q1000-EX Shimadzu and GCMS 5988-A HP spectrometers, the ionizing voltage was 70 eV. Elemental analyses were carried out by the Microanalytical Center of Cairo University, Giza, Egypt. Antitumor activity was evaluated by the National Institute of Cancer, Biology Department, Cairo University, Egypt. Antimicrobial activity was carried out at the Regional Center for Mycology and Biotechnology at Al-Azhar University, Cairo, Egypt. 3-Acetyl-4-(4-nitrophenyl)-1-aryl-1*H*-pyrazoles **1a,b** [26] and hydrazonoyl halides [38,39,40,41,42,43] **18a-c** were prepared following literature methods.

### 3.2. Synthesis of 3-[E-3-(N,N-dimethylamino)acryloyl]-4-(4-nitrophenyl)-1-aryl-1H-pyrazoles **2a,b**

A mixture of 3-acetyl-4-(4-nitrophenyl)-1-aryl-1*H*-pyrazoles (**1a **or **1b**) (0.01 mol) and dimethyl-formamide dimethylacetal (DMF-DMA) (5 mL) was refluxed for 10 hours. After cooling, methanol was added and the solid product was collected by filtration and crystallized from ethanol. The physical constants and the spectral data are shown below.

*3-[E-3-(N,N-dimethylamino)acryloyl]-4-(4-nitrophenyl)-1-phenyl-1H-pyrazole* (**2a**). Yellow crystals, (2.89 g, 80%), m.p. 132–134 °C; IR (KBr) υ = 1,642 (CO) cm^−1^; ^1^H-NMR (CDCl_3_) *δ* = 2.82 (s, 3H, CH_3_), 3.00 (s, 3H, CH_3_), 5.88 (d, 1H, *J* = 13 Hz, CH=), 7.27 (d, 2H, *J* = 8 Hz, Ar-H), 7.67 (d, 1H, *J* = 13 Hz, CH=), 7.35–7.68 (m, 5H, Ar-H), 8.05 (d, 2H, *J* = 8 Hz, Ar-H), 8.90 (s, 1H, pyrazole-H-5) ppm; MS, *m/z* (%) 362 (M^+^, 25), 292 (30), 264 (20), 122 (15), 98 (40), 77 (100), 70 (40). Anal. Calcd. for C_20_H_18_N_4_O_3_ (362.14): C, 66.29; H, 5.01; N, 15.46. Found: C, 66.18; H, 4.93; N, 15.58%.

*3-[E-3-(N,N-dimethylamino)acryloyl]-1-(4-methylphenyl)-4-(4-nitrophenyl)-1H-pyrazole* (**2b**). Yellow crystals, (3.31 g, 88%), m.p. 148–150 °C; IR (KBr) υ = 1647 (CO) cm^−1^; ^1^H-NMR (DMSO-d_6_) *δ* = 2.37 (s, 3H, Ar-CH_3_), 2.89 (s, 3H, CH_3_), 3.13 (s, 3H, CH_3_), 5.88 (d, 1H, *J* = 13 Hz, CH=), 7.35 (d, 2H, *J* = 8 Hz, Ar-H), 7.66 (d, 1H, *J* = 13 Hz, CH=), 7.82 (d, 2H, *J* = 8 Hz, Ar-H), 7.91 (d, 2H, *J* = 8 Hz, Ar-H), 8.21 (d, 2H, *J* = 8 Hz, Ar-H), 8.92 (s, 1H, pyrazole-H-5) ppm; MS, *m/z* (%) 376 (M^+^, 25), 306 (40), 278 (20), 98 (40), 92 (85), 77 (100), 70 (40). Anal. Calcd. for C_21_H_20_N_4_O_3_ (376.15): C, 67.01; H, 5.36; N, 14.88. Found: C, 67.12; H, 5.23; N, 14.71%.

### 3.3. Reactions of 3-[E-3-(N,N-dimethylamino)acryloyl]-4-(4-nitrophenyl)-1-aryl-1H-pyrazoles **2a,b** with Active Methylene Compounds

To a solution of **2a **or **2b** (1 mmol) and 2,4-pentanedione (**3a**) or ethyl 3-oxobutanoate (**3b**) (1 mmol) in acetic acid (20 mL) was added ammonium acetate (0.156 g, 2 mmol). The reaction mixture was heated under reflux for 5 hours. After cooling, the reaction mixture was poured onto ice and the solid product was collected by filtration and crystallized from an ethanol/dioxane mixture (1:1). The physical constants, together with the spectral data for products **5a-d**, are shown below.

*3-Acetyl-2-methyl-6-[4-(4-nitrophenyl)-1-phenyl-1H-pyrazol-3-yl]pyridine* (**5a**). Yellow crystals, (0.34 g, 85%), m.p. 318–320 °C; IR (KBr) υ = 1,691 (CO) cm^−1^; ^1^H-NMR (DMSO-d_6_) *δ* = 2.32 (s, 3H, CH_3_), 2.42 (s, 3H, COCH_3_), 7.35 (d, 2H, *J* = 8 Hz, Ar-H), 7.55 (d, 1H, *J* = 8 Hz, pyridyl H-4), 7.67 (d, 1H, *J* = 8 Hz, pyridyl H-5), 7.71–8.20 (m, 5H, Ar-H), 8.26 (d, 2H, *J* = 8 Hz, Ar-H), 9.01 (s, 1H, pyrazole-H-5) ppm; MS, *m/z* (%) 398 (M^+^, 60), 355 (30), 122 (25), 77 (100). Anal. Calcd. for C_23_H_18_N_4_O_3_ (398.14): C, 69.34; H, 4.55; N, 14.06. Found: C, 69.27; H, 4.68; N, 14.11%.

*3-Acetyl-2-methyl-6-[4-(4-nitrophenyl)-1-(4-methylphenyl)-1H-pyrazol-3-yl]pyridine* (**5b**). Yellow crystals, (0.35 g, 85%), m.p. 322–325 °C; IR (KBr) υ = 1,692 (CO) cm^−1^; ^1^H-NMR (DMSO-d_6_) *δ* = 2.32 (s, 3H, CH_3_), 2.38 (s, 3H, Ar-CH_3_), 2.44 (s, 3H, COCH_3_), 7.37 (d, 2H, *J* = 8 Hz, Ar-H), 7.53 (d, 1H, *J* = 8 Hz, pyridyl H-4), 7.61 (d, 1H, *J* = 8 Hz, pyridyl H-5), 7.76–8.26 (m, 4H, Ar-H), 8.29 (d, 2H, *J* = 8 Hz, Ar-H), 9.11 (s, 1H, pyrazole H-5) ppm; MS, *m/z* (%) 412 (M^+^, 75), 369 (30), 122 (25), 91 (50), 77 (100). Anal. Calcd. for C_24_H_20_N_4_O_3_ (412.15): C, 69.89; H, 4.89; N, 13.58. Found: C, 69.77; H, 4.78; N, 13.41%.

*Ethyl 2-methyl-6-[4-(4-nitrophenyl)-1-phenyl-1H-pyrazol-3-yl]nicotinate* (**5c**). Pale yellow crystals, (0.35 g, 82%), m.p. 180–182 °C; IR (KBr) υ = 1,706 (CO) cm^−1^; ^1^H-NMR (DMSO-d_6_) *δ* = 1.31 (t, 3H, *J* = 7 Hz, CH_3_), 2.38 (s, 3H, CH_3_), 4.35 (q, 2H, *J* = 7 Hz, CH_2_), 7.36 (d, 2H, *J* = 8 Hz, Ar-H), 7.52 (d, 1H, *J* = 8 Hz, pyridyl H-4), 7.62 (d, 1H, *J* = 8 Hz, pyridyl H-5), 7.81-8.20 (m, 5H, Ar-H), 8.28 (d, 2H, *J* = 8 Hz, Ar-H), 8.97 (s, 1H, pyrazole H-5) ppm; MS, *m/z* (%) 428 (M^+^, 60), 355 (30), 122 (25), 77 (100). Anal. Calcd. for C_24_H_20_N_4_O_4_ (428.15): C, 67.28; H, 4.71; N, 13.08. Found: C, 67.19; H, 4.62; N, 13.16%.

*Ethyl 2-methyl-6-[4-(4-nitrophenyl)-1-(4-methylphenyl)-1H-pyrazol-3-yl]nicotinate* (**5d**). Pale yellow crystals, (0.37 g, 85%), m.p. 186–188 °C; IR (KBr) υ = 1,709 (CO) cm^−1^; ^1^H-NMR (DMSO-d_6_) *δ* = 1.33 (t, 3H, *J* = 7 Hz, CH_3_), 2.38 (s, 3H, CH_3_), 2.41 (s, 3H, Ar-CH_3_), 4.39 (q, 2H, *J* = 7 Hz, CH_2_), 7.38 (d, 2H, *J* = 8 Hz, Ar-H), 7.51 (d, 1H, *J* = 8 Hz, pyridyl H-4), 7.59 (d, 1H, *J* = 8 Hz, pyridyl H-5), 7.72–8.10 (m, 4H, Ar-H), 8.29 (d, 2H, *J* = 8 Hz, Ar-H), 8.99 (s, 1H, pyrazole H-5) ppm; MS, *m/z* (%) 442 (M^+^, 50), 369 (30), 122 (25), 91 (100), 77 (60). Anal. Calcd. for C_25_H_22_N_4_O_4_ (442.16): C, 67.86; H, 5.01; N, 12.66. Found: C, 67.74; H, 4.92; N, 12.56%.

### 3.4. Reactions of 3-[E-3-(N,N-dimethylamino)acryloyl]-4-(4-nitrophenyl)-1-aryl-1H-pyrazoles **2a,b** with Hydrazine Hydrate

To a solution of the enaminone (**2a** or **2b**) (1 mmol) in ethanol (10 mL) was added hydrazine hydrate (1 mL) and the mixture was heated under reflux for 5 hours. The reaction mixture was acidified by HCl/ice mixture and the formed product was filtered and crystallized from ethanol.

*4-(4-Nitrophenyl)-1-phenyl-1H,1'H-3,3'-bipyrazole* (**8a**). Yellow crystals, (0.30 g, 90%), m.p. 200–202 °C; IR (KBr) υ = 3,246 (NH) cm^–1^; ^1^H-NMR (DMSO-d_6_) *δ* = 7.25 (d, 2H, *J* = 8 Hz, Ar-H), 7.35–7.97 (m, 5H, Ar-H), 7.53 (d, 1H, *J* = 7.5 Hz*,* pyrazole H-4), 7.58 (d, 1H, *J* = 7.5 Hz*,* pyrazole H-5), 8.23 (d, 2H, *J* = 8 Hz, Ar-H), 9.01 (s, 1H, pyrazole H-5), 13.00 (D_2_O-exchangeable) (s, 1H, NH) ppm; MS, *m/z* (%) 331 (M^+^, 60), 284 (20), 122 (25), 77 (100). Anal. Calcd. for C_18_H_13_N_5_O_2 _(331.11): C, 65.25; H, 3.95; N, 21.14. Found: C, 65.37; H, 3.88; N, 21.21%.

*4-(4-Nitrophenyl)-1-(4-methylphenyl)-1H,1'H-3,3'-bipyrazole* (**8b**). Yellow crystals, (0.31 g, 90%), m.p. 172–174 °C; IR (KBr) υ = 3,226 (NH) cm^−1^; ^1^H-NMR (DMSO-d_6_) *δ* = 2.36 (s, 3H, Ar-CH_3_), 7.23 (d, 2H, *J* = 8 Hz, Ar-H), 7.25–7.91 (m, 4H, Ar-H), 7.51 (d, 1H, *J* = 7.5 Hz*,* pyrazole H-4), 7.56 (d, 1H, *J* = 7.5 Hz*,* pyrazole H-5), 8.20 (d, 2H, *J* = 8 Hz, Ar-H), 9.00 (s, 1H, pyrazole H-5), 12.97 (D_2_O-exchangeable) (s, 1H, NH) ppm; MS, *m/z* (%) 345 (M^+^, 70), 299 (20), 122 (25), 91 (100), 77 (80). Anal. Calcd. for C_19_H_15_N_5_O_2 _(345.12): C, 66.08; H, 4.38; N, 20.28. Found: C, 66.12; H, 4.46; N, 20.18%.

### 3.5. Reactions of 3-[E-3-(N,N-dimethylamino)acryloyl]-4-(4-nitrophenyl)-1-aryl-1H-pyrazoles **2a,b** with Hydroxylamine Hydrochloride

Hydroxylamine hydrochloride (0.07 g, 1 mmol) was added to a mixture of enaminone **2a** or **2b** (1 mmol) and anhydrous potassium carbonate (0.5 g) in absolute ethanol (20 mL). The mixture was heated under reflux for 5 hours and poured onto water. The solid product was filtered and crystallized from ethanol.

*3-[4-(4-Nitrophenyl)-1-phenyl-1H-pyrazol-3-yl]isoxazole* (**9a**). Yellow crystals, (0.25 g, 75%), m.p. 160–162 °C; IR (KBr) υ = 1,600 (C=N), cm^−1^; ^1^H-NMR (DMSO-d_6_) *δ* = 6.78 (d, 1H, *J* = 5 Hz, isoxazole H-4), 7.29 (d, 2H, *J* = 8 Hz, Ar-H), 7.36–8.21 (m, 5H, Ar-H), 8.48 (d, 2H, *J* = 8 Hz, Ar-H), 8.72 (d, 1H, *J* = 5 Hz, isoxazole H-5), 9.05 (s, 1H, pyrazole H-5) ppm; MS, *m/z* (%) 332 (M^+^, 60), 286 (20), 122 (25), 77 (100). Anal. Calcd. for C_18_H_12_N_4_O_3 _(332.09): C, 65.06; H, 3.64; N, 16.86. Found: C, 65.17; H, 3.58; N, 16.71%.

*3-[4-(4-Nitrophenyl)-1-(4-methylphenyl)-1H-pyrazol-3-yl]isoxazole* (**9b**). Yellow crystals, (0.26 g, 75%), m.p. 170–172 °C; IR (KBr) υ = 1,601 (C=N), cm^−1^; ^1^H-NMR (DMSO-d_6_) *δ* = 2.37 (s, 3H, Ar-CH_3_), 6.78 (d, 1H, *J* = 5 Hz*,* isoxazole H-4), 7.35 (d, 2H, *J* = 8 Hz, Ar-H), 7.39–8.32 (m, 4H, Ar-H), 8.68 (d, 2H, *J* = 8 Hz, Ar-H), 8.79 (d, 1H, *J* = 5 Hz, isoxazole H-5), 9.04 (s, 1H, pyrazole H-5) ppm; MS, *m/z* (%) 346 (M^+^, 50), 300 (20), 122 (25), 91 (100), 77 (60). Anal. Calcd. for C_19_H_14_N_4_O_3 _(346.11): C, 65.89; H, 4.07; N, 16.18. Found: C, 65.77; H, 3.98; N, 16.11%.

### 3.6. Reactions of 3-[E-3-(N,N-dimethylamino)acryloyl]-4-(4-nitrophenyl)-1-aryl-1H-pyrazoles **2a,b** with 3-amino-1H-[1,2,4]triazole

A mixture of enaminone **2a** or **2b** (1 mmol) and 3-amino-1*H*-[1,2,4]triazole (0.085 g, 1 mmol), in glacial acetic acid (20 mL), was refluxed for 5 hours. The solid that formed was filtered off, and crystallized from dioxane to afford compounds **12a**,**b**. 

*5-[4-(4-Nitrophenyl)-1-phenyl-1H-pyrazol-3-yl][1,2,4]triazolo[4,3-a]pyrimidine* (**12a**). Yellow crystals, (0.32 g, 85%), m.p. 290–292 °C; IR (KBr) υ = 1,596 (C=N) cm^−1^; ^1^H-NMR (DMSO-d_6_) *δ* = 7.45 (d, 2H, *J* = 8 Hz, Ar-H), 7.59–8.01 (m, 5H, Ar-H), 7.71 (d, 1H, *J* = 5 Hz, pyrimidine H-5), 8.11 (d, 2H, *J* = 8 Hz, Ar-H), 8.47 (s, 1H, triazole H-5), 9.01 (s, 1H, pyrazole H-5), 9.34 (d, 1H, *J* = 5 Hz, pyrimidine H-4) ppm; MS, *m/z* (%) 383 (M^+^, 50), 337 (40), 122 (25), 77 (100). *Anal*. Calcd. for C_20_H_13_N_7_O_2 _(383.11): C, 62.66; H, 3.42; N, 25.58. Found: C, 62.77; H, 3.58; N, 25.71%.

*5-[4-(4-Nitrophenyl)-1-(4-methylphenyl)-1H-pyrazol-3-yl][1,2,4]triazolo[4,3-a]pyrimidine* (**12b**). Yellow crystals, (0.34 g, 85%), m.p. 310–312 °C; IR (KBr) υ = 1,598 (C=N) cm^−1^; ^1^H-NMR (DMSO-d_6_) *δ* = 2.41 (s, 3H, Ar-CH_3_), 7.37 (d, 2H, *J* = 8 Hz, Ar-H), 7.49–8.01 (m, 4H, Ar-H), 7.74 (d, 1H, *J* = 5 Hz, pyrimidine H-5), 8.16 (d, 2H, *J* = 8 Hz, Ar-H), 8.49 (s, 1H, triazole H-5), 9.03 (s, 1H, pyrazole H-5), 9.36 (d, 1H, *J* = 5 Hz, pyrimidine H-4) ppm; MS, *m/z* (%) 397 (M^+^, 50), 351 (40), 122 (25), 91 (70), 77 (100). *Anal*. Calcd. for C_21_H_15_N_7_O_2 _(397.13): C, 63.47; H, 3.80; N, 24.67. Found: C, 63.58; H, 3.62; N, 24.77%.

### 3.7. Coupling of 3-[E-3-(N,N-dimethylamino)acryloyl]-4-(4-nitrophenyl)-1-aryl-1H-pyrazoles **2a,b** with diazonium salt of 3-amino-1H-[1,2,4]triazole

To a cold solution of enaminone **2a** or **2b **(1 mmol) in pyridine (25 mL) was added the heterocyclic diazonium salt [prepared by diazotizing 3-amino-1*H*-[1,2,4]triazole (0.085 g, 1 mmol) dissolved in concentrated nitric acid (2 mL) with a solution of sodium nitrite (0.07 g, 1 mmol) in water (2 mL)]. After complete addition of the diazonium salt, the reaction mixture was stirred for a further 30 min in an ice bath, and then poured onto ice/HCl mixture. The solid precipitated was filtered off, washed with water, dried and crystallized from ethanol/dioxane mixture to give the respective products **14a** and **14b**.

*[4-(4-Nitrophenyl)-1-phenyl-1H-3-pyrazolyl]carbonyl[1,2,4]triazolo[3,4-c][1,2,4]triazine* (**14a**). Pale yellow crystals, (0.32 g, 80%), m.p. 290–292 °C; IR (KBr) υ = 1,662 (CO) cm^−1^; ^1^H-NMR (DMSO-d_6_) *δ* = 7.43 (d, 2H, *J* = 8 Hz, Ar-H), 7.56–7.96 (m, 5H, Ar-H), 7.77 (s, 1H, triazine H-5), 8.22 (d, 2H, *J* = 8 Hz, Ar-H), 8.48 (s, 1H, triazole H-5), 9.17 (s, 1H, pyrazole H-5) ppm; MS, *m/z* (%) 412 (M^+^, 50), 292 (20), 122 (50), 77 (100). Anal. Calcd. for C_20_H_12_N_8_O_3 _(412.10): C, 58.25; H, 2.93; N, 27.17. Found: C, 58.37; H, 3.02; N, 27.31%.

*[4-(4-Nitrophenyl)-1-(4-methylphenyl)-1H-3-pyrazolyl]{[1,2,4]triazolo[3,4-c][1,2,4]triazin-6-yl}-methanone* (**14b**). Pale yellow crystals, (0.34 g, 80%), m.p. 198–200 °C; IR (KBr) υ = 1,664 (CO) cm^−1^; ^1^H-NMR (DMSO-d_6_) *δ* = 2.39 (s, 3H, Ar-CH_3_), 7.46 (d, 2H, *J* = 8 Hz, Ar-H), 7.51–7.99 (m, 4H, Ar-H), 7.78 (s, 1H, triazine H-5), 8.26 (d, 2H, *J* = 8 Hz, Ar-H), 8.49 (s, 1H, triazole H-5), 9.13 (s, 1H, pyrazole H-5) ppm; MS, *m/z* (%) 426 (M^+^, 50), 306 (20), 148 (60), 122 (50), 77 (100). Anal. Calcd. for C_21_H_14_N_8_O_3 _(426.12): C, 59.15; H, 3.31; N, 26.28. Found: C, 59.39; H, 3.22; N, 26.38%.

### 3.8. Synthesis of 5-[4-(4-nitrophenyl)-1-phenyl-1H-pyrazol-3-yl]-2-thioxo-2,3-dihydro-1H-pyrido[2,3-d]pyrimidin-4-one (17)

A mixture of 3-[*E*-3-(*N*,*N*-dimethylamino)acryloyl]-4-(4-nitrophenyl)-1-phenyl-1*H*-pyrazole (**2a**) (1.81 g, 5 mmol) and 6-amino-2-thioxo-2,3-dihydropyrimidin-4(1*H*)-one (**15**, 0.715 g, 5 mmol) in acetic acid (20 mL) was refluxed for 6 hours. The reaction mixture was cooled and diluted with methanol and the solid product was collected by filtration and recrystallized from dioxane to give **17**. Yellow crystals (0.35 g, 80%), m.p. 310–313 °C; IR (KBr) υ = 3,261, 3,245 (2 NH), 1,677 (CO), cm^−1^; ^1^H-NMR (DMSO-d_6_) *δ* = 7.42 (d, 2H, *J* = 8 Hz, Ar-H), 7.49–8.20 (m, 5H, Ar-H), 8.24 (d, 2H, *J* = 8 Hz, Ar-H), 8.29 (d, 1H, *J* = 7 Hz*,* pyridine-H), 8.48 (d, 1H, *J* = 7 Hz*,* pyridine-H), 9.05 (s, 1H, pyrazole H-5), 12.62 (s, 1H, NH), 13.14 (s, 1H, NH) ppm; MS, *m/z* (%) 442 (M^+^, 40), 396 (20), 122 (40), 77 (100). Anal. Calcd. for C_22_H_14_N_6_O_3_S (442.08): C, 59.72; H, 3.19; N, 18.99; S, 7.25. Found: C, 59.81; H, 3.14; N, 19.04; S, 7.20%.

### 3.9. Synthesis of pyrido[2,3-d][1,2,4]triazolo[4,3-a]pyrimidin-5-one derivatives 21a-c

To a mixture of equimolar amounts of **17** and the appropriate hydrazonoyl chlorides **18a-c** (1 mmol) in dioxane (15 mL) was added triethylamine (0.14 mL, 1 mmol). The reaction mixture was refluxed until all of the starting materials have disappeared and hydrogen sulfide gas ceased to evolve (6 hours, monitored by TLC). The solvent was evaporated and the residue was triturated with methanol. The solid that formed was filtered and crystallized from methanol/dioxane mixture to give compounds **21a-c**.

*3-Acetyl-6-[4-(4-nitrophenyl)-1-phenyl-1H-pyrazol-3-yl]-1-phenyl-1,5-dihydropyrido[2,3-d][1,2,4] triazolo[4,3-a]pyrimidin-5-one* (**21a**). Yellow crystals, (0.45 g, 80%), m.p. 280–282 °C; IR (KBr) υ = 1,707, 1,650 (2 CO), cm^−1^; ^1^H-NMR (DMSO-d_6_) δ = 2.84 (s, 3H, COCH_3_), 7.26 (d, 2H, J = 8 Hz, Ar-H), 7.39–7.85 (m, 10H, Ar-H), 8.03 (d, 1H, J = 7 Hz, pyridine-H), 8.27 (d, 2H, J = 8 Hz, Ar-H), 8.69 (d, 1H, J = 7 Hz, pyridine-H), 9.05 (s, 1H, pyrazole H-5) ppm; ^13^C-NMR (DMSO-d_6_) δ = 31.3, 119.8, 121.7, 122.4, 123.3, 124.5, 125.3, 127.4, 128.5, 129.1, 129.8, 131.2, 139.4, 142.5, 143.9, 146.8, 147.8, 148.1, 148.8, 152.1, 153.8, 155.3, 159.5, 164.0, 176 ppm; MS, m/z (%) 568 (M^+^, 25), 525 (20), 497 (40), 122 (30), 77 (100). Anal. Calcd. for C_31_H_20_N_8_O_4_ (568.16): C, 65.49; H, 3.55; N, 19.71. Found: C, 65.34; H, 3.42; N, 19.64%.

*Ethyl 5-oxo-6-[4-(4-nitrophenyl)-1-phenyl-1H-pyrazol-3-yl]-1-phenyl-1,5-dihydropyrido[2,3-d] [1,2,4]triazolo[4,3-a]pyrimidine-3-carboxylate* (**21b**). Yellow crystals, (0.47 g, 80%), m.p. 250–253 °C; IR (KBr) υ = 1,719, 1,645 (2 CO), cm^−1^; ^1^H-NMR (DMSO-d_6_) δ = 1.45 (t, J = 7 Hz, 3H, CH_3_,), 4.57 (q, J = 7 Hz, 2H, CH_2_,), 7.26 (d, 2H, J = 8 Hz, Ar-H), 7.27–7.81 (m, 10H, Ar-H), 8.16 (d, 1H, J = 7 Hz, pyridine-H), 8.21 (d, 2H, J = 8 Hz, Ar-H), 8.62 (d, 1H, J = 7 Hz, pyridine-H), 9.07 (s, 1H, pyrazole H-5) ppm; ^13^C-NMR (DMSO-d_6_) δ = 31.6, 35.8, 118.9, 120.7, 122.4, 123.3, 124.6, 125.7, 127.2, 128.5, 129.3, 129.9, 131.2, 139.4, 142.5, 143.7, 146.8, 147.9, 148.2, 148.8, 152.1, 153.8, 155.3, 159.5, 163.4, 177 ppm; MS, m/z (%) 598 (M^+^, 25), 525 (40), 479 (40), 122 (30), 77 (100). Anal. Calcd. for C_32_H_22_N_8_O_5_ (598.17): C, 64.21; H, 3.70; N, 18.72. Found: C, 64.34; H, 3.62; N, 18.62%.

*N3,1-Diphenyl-5-oxo-6-[4-(4-nitrophenyl)-1-phenyl-1H-pyrazol-3-yl]-1,5-dihydropyrido[2,3-d] [1,2,4]triazolo[4,3-a]pyrimidine-3-carboxamide* (**21c**). Yellow crystals, (0.48 g, 75%), m.p. 325−327 °C; IR (KBr) υ = 3,388 (NH), 1,697, 1,651 (2 CO), cm^−1^; ^1^H-NMR (DMSO-d_6_) δ = 7.23 (d, 2H, J = 8 Hz, Ar-H), 7.39–8.15 (m, 15H, Ar-H), 8.01 (d, 1H, J = 7 Hz, pyridine-H), 8.24 (d, 2H, J = 8 Hz, Ar-H), 8.62 (d, 1H, J = 7 Hz, pyridine-H), 9.01 (s, 1H, pyrazole H-5), 10.92 (s, 1H, NH) ppm; ^13^C-NMR (DMSO-d_6_) δ = 111.8, 119.8, 120.7, 121.4, 121.7, 122.4, 123.3, 124.5, 125.3, 125.9, 127.4, 128.5, 129.1, 129.8, 131.2, 139.4, 142.5, 143.9, 146.8, 147.8, 148.1, 148.8, 152.1, 153.8, 155.3, 159.5, 161.9, 168 ppm; MS, m/z (%) 645 (M^+^, 25), 525 (40), 122 (20), 77 (100). Anal. Calcd. for C_36_H_23_N_9_O_4_ (645.19): C, 66.97; H, 3.59; N, 19.53. Found: C, 66.84; H, 3.46; N, 19.61%.

### 3.10. Agar diffusion well method to determine the antimicrobial activity

The microorganism inoculums were uniformly spread using sterile cotton swab on a sterile Petri dish Malt extract agar (for fungi) and nutrient agar (for bacteria). One hundred μL of each sample was added to each well (10 mm diameter holes cut in the agar gel, 20 mm apart from one another). The systems were incubated for 24–48 h at 37 °C (for bacteria) and at 28 °C (for fungi). After incubation, the microorganism's growth was observed. Inhibition of the bacterial and fungal growth were measured in mm. Tests were performed in triplicate [44].

### 3.11. Cytotoxic activity

The method applied is similar to that reported by Skehan *et al.* [45] using Sulfo-Rhodamine-B stain (SRB). Cells were plated in 96-multiwill plate (10^4 ^cells/well) for 24 h before treatment with the tested compounds to allow attachment of cell to the wall of the plate. Different concentrations of the compound under test (0, 2.5, 5, and 10 µg/mL) were added to the cell monolayer in triplicate wells individual dose, monolayer cells were incubated with the compounds for 48 h at 37 °C and in atmosphere of 5% CO_2_. After 48 h, cells were fixed, washed and stained with SRB stain, excess stain was washed with acetic acid and attached stain was recovered with *tris*-EDTA buffer, color intensity was measured in an ELISA reader. The relation between surviving fraction and drug concentration is plotted to get the survival curve of each tumor cell line after the specified compound. The response parameter calculated was the IC_50_ value, which corresponds to the compound concentration causing 50% mortality in net cells (Figures 3-10). 

## 4. Conclusions

In this study, synthetic routes to a wide variety of azoles, fused azoles, and azines at the 3-position of *N*-arylpyrazole ring were developed using novel enaminones as building blocks. Moreover, some of the newly synthesized products were tested as antitumor and antimicrobial agents and the results obtained were promising. 
